# Role of Caveolin-1 in Diabetes and Its Complications

**DOI:** 10.1155/2020/9761539

**Published:** 2020-01-27

**Authors:** Dania Haddad, Ashraf Al Madhoun, Rasheeba Nizam, Fahd Al-Mulla

**Affiliations:** Genetics and Bioinformatics Department, Dasman Diabetes Institute, Kuwait City, Kuwait

## Abstract

It is estimated that in 2017 there were 451 million people with diabetes worldwide. These figures are expected to increase to 693 million by 2045; thus, innovative preventative programs and treatments are a necessity to fight this escalating pandemic disorder. Caveolin-1 (CAV1), an integral membrane protein, is the principal component of caveolae in membranes and is involved in multiple cellular functions such as endocytosis, cholesterol homeostasis, signal transduction, and mechanoprotection. Previous studies demonstrated that CAV1 is critical for insulin receptor-mediated signaling, insulin secretion, and potentially the development of insulin resistance. Here, we summarize the recent progress on the role of CAV1 in diabetes and diabetic complications.

## 1. Introduction

Caveolae are specialized, bulb-shaped, 50-100 nm wide cholesterol-rich lipid rafts found in the plasma membrane of most cell types [1]. They were once thought to be simple membrane structures but are now considered to be a more complex bona fide organelle. Caveolar density differs with cell type; caveolae are abundant in adipocytes, vascular endothelial cells, smooth muscle cells, fibroblasts, and epithelial cells. In adipocytes, caveolae constitute one-third of the membrane area [2]. Caveolae increase significantly the cellular surface area and are implicated in several essential cellular functions such as endocytosis, transcytosis, maintenance of plasma membrane integrity, lipid homeostasis, signal transduction, and mechanoprotection [3]. Despite caveolae's diverse functions, their physiological role in different cell types is not fully understood.

Caveolae are mostly made up of caveolins (1/2/3); a ∼22 kDa protein termed caveolin-1 (CAV1) oligomerizes in the endoplasmic reticulum (∼14–16 monomers form a ∼350 kDa oligomer) [4]. At the Golgi body, CAV1 oligomers interact with cholesterol molecules and then the newly formed complexes are transported to the cellular membrane where additional cytosolic adaptor proteins named avins contribute to caveolar formation. CAV1 mediates the recruitment of Cavin proteins (CAVIN1/2/3/4) to the caveolae [5–7]. Each caveola has around 140-150 CAV1 molecules [8]. Caveolar vesicles are also highly organized and enriched in saturated phospholipids, sphingolipids, plasmenylethalomines, and cholesterol [9]. Apart from caveolae, oligomerized CAV1 scaffolds exist in the plasma membrane and partake in CAV1-dependent signal regulation as well [10]. CAV1 molecules reside not only in the cell membrane but also in other membranous structures such as mitochondria and secretory vesicles (e.g., insulin granules) [11]. Caveolins interact with various signaling proteins via their N-termini termed the caveolin-scaffolding domain (CSD, 82-101 aa) [12]. Transport of caveolae-mediated vesicles (non-clathrin-mediated) follows predominantly the transcytotic route to transport solutes from the blood to underlying tissues [5]. Src kinases mediate CAV1 phosphorylation at Tyrosine 14 (P-Y14) and of dynamin-2, both required for caveolae-mediated endocytosis and trafficking [13]. Depletion of CAV1 and the resultant reduction in the number of caveolae have been recently associated with a broad range of disease states such as cancer and cardiovascular and pulmonary diseases [14–16].

In order to define the relationship between CAV1 and diabetes, in this review, we cover the state-of-the-art development and progress on CAV1 and diabetes, altogether describing the role of CAV1 in insulin secretion, insulin signaling, insulin resistance, oxidative stress, diabetic complications, diabetic drug effects on CAV1, and future therapeutic perspectives in the hope of supporting clinical applications of CAV1.

## 2. Role of Caveolin-1 in Insulin Secretion and Development of Diabetes

In pancreatic *β*-cells, CAV1 plays a role in insulin receptor- (IR-) mediated signaling, insulin secretion, and possibly in diabetes. Under physiological low glucose conditions, CAV1 forms a complex with insulin granule proteins including the Rho GTPase cell division cycle 42 (cdc42), vesicle-associated membrane protein 2 (VAMP2), and the guanine nucleotide exchange factor 7 (*β*PIX). Glucose stimulus mediates CAV1 dissociation and complex disassembly and promotes insulin secretion [17]. In pancreatic cell lines INS-1 and MIN6, CAV1 siRNA knockdown resulted in a significant increase in insulin secretion under physiological glucose levels [17, 18].

Further studies indicated that CAV1 depletion *in vivo* enhances insulin secretion. Relative to littermate controls, CAV1 knockout (KO) mice suffer from hyperinsulinemia under fasting conditions and when fed with high-fat diet (HFD) [19]. Recently, the mechanisms associated with CAV1 silencing and *β*-cell homeostasis, survival, and insulin secretion have been revealed. Zeng et al. found that in pancreatic *β*-cells, lipotoxicity is moderated when CAV1 is depleted due to the activation of AKT (RAC-alpha serine threonine protein kinase) and ERK1/2 (extracellular-signal-regulated kinase) signaling pathways, which in turn downregulate the expression of cell cycle arrest proteins and upregulate the expression of cell cycle activators [20].

**Figure 1:**
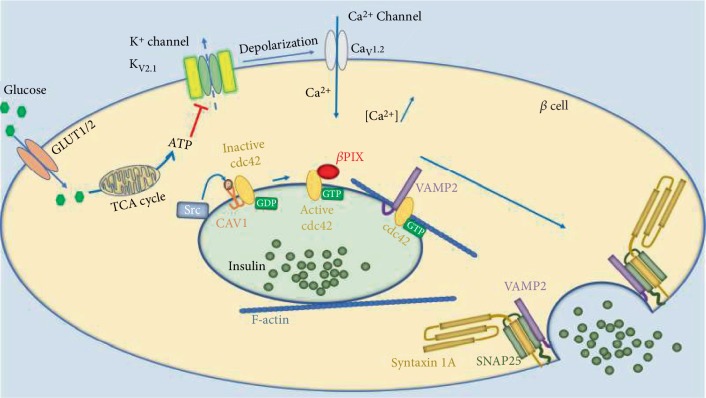
Insulin secretion mechanism and the role of CAV1. At high glucose conditions, the increased ATP/ADP ratio results in the closure of the ATP-sensitive K^+^ channel K_v_2.1, which in turn prompts the opening of the voltage-dependent Ca^2+^ channel Ca_v_1.2. The increased cytoplasmic Ca^2+^ triggers the activation of exocytotic machinery. The process is initiated by the dissociation of the CAV1-cdc42-GDP complex through CAV1^Tyr14^ phosphorylation. The released inactive cdc42-GDP binds to *β*PIX resulting in an active cdc42-GTP, which interacts with VAMP2-bound insulin secretory granules. These vesicles are then targeted to fusion with the plasma membrane through the interaction between cdc42, VAMP2, F-actin, Syntaxin 1A, and SNAP-25 modulations. Abbreviations: *β* cell—pancreatic *β* cell line; GLUT—glucose transporter; TCA cycle—tricarboxylic acid cycle or Krebs cycle; cdc42—cell division cycle 42; *β*PIX—guanine nucleotide exchange factor 7; VAMP2—vesicle-associated membrane protein 2; F-actin—filamentous actin; SNAP-25—synaptosomal-associated protein 25.

The molecular mechanism underlying insulin secretion is described in Figure 1 (best reviewed in [21]). At high glucose conditions, the increased ATP/ADP ratio results in the closure of the ATP-sensitive K^+^ channel K_v_2.1, which in turn prompts the opening of the voltage-dependent Ca^2+^ channel Ca_v_1.2 [22]. The increase in cytoplasmic Ca^2+^ ion concentration triggers the activation of exocytotic machinery [23]. The process is initiated by the dissociation of the CAV1-cdc42-GDP complex through active Src kinase-mediated CAV1 P-Y14 phosphorylation [17, 24, 25]. The released inactive cdc42-GDP binds to *β*PIX resulting in conformational changes that activate cdc42-GTP [17, 26]. On a side note, it has been proposed that YES, a Src family kinase, might be the kinase that is either phosphorylating CAV1 or *β*PIX and thus plays an important role in insulin secretion in MIN6 cells. Cdc42 is now active, and it interacts with insulin secretory granule-bound VAMP2 molecules, which are then targeted to fusion with the plasma membrane through the indirect interaction between cdc42, VAMP2, F-actin (filamentous actin), Syntaxin 1A, and SNAP-25 (synaptosomal-associated protein 25) modulations [27, 28].

In sum, CAV1 facilitates insulin secretion through at least interacting with cdc42 and being an integral part of the insulin secretion vesicles.

## 3. Role of Caveolin-1 in Insulin Signaling and Insulin Resistance

Caveolae have an abundant number of intramembrane signaling proteins with lipid modification docked within them, allowing signaling cascade interaction [29]. CAV1 is central to the adjustment of these processes. It recruits and regulates various signaling proteins through the interaction of the CSD. CAV1 can negatively modulate signal transduction pathways such as those involving transforming growth factor-beta receptor 1, nitric oxide synthases, G proteins, protein *kinase* A, ERK1/2, and phosphatidylinositol-3-kinase- (PI3K-) AKT [30–32] or enhance the signal of downstream effector proteins such as IR, estrogen receptor, protein lipase C, and protein lipase D [19, 33–36].

### 3.1. Role of Caveolin-1 in Insulin Signaling

Multiple lines of accumulated evidence indicate a vital role of caveolae in regulating not only insulin secretion but also insulin signaling [33]. In the plasma membrane, structural studies showed that IR is mainly localized and highly enriched in caveolae with very little receptors outside of caveolae [37]. Furthermore, rat hepatocytes fed with HFD had caveolae that contained more IR than those fed with regular chow [38]. In pancreatic *β* cells, IR signaling was impaired by cholesterol depletion or by expression of a dominant-negative CAV1 mutant, further demonstrating that CAV1 plays an essential role for proper insulin response. Similarly, CAV1-/- mice that lack caveolae in all tissues (except tissues expressing caveolin-3) have a reduced amount of IR in adipocytes and exhibit the same characteristics observed in humans suffering from congenital generalized human Lipodystrophy type 3 (CGL3). CAV1-null mice on normal diet show no response to insulin stimulation due to the IR protein levels decreased by 90% even with normal mRNA expression, which suggest that CAV1 stabilizes IR in the plasma membrane [19].

In fact, CAV1 was found to directly interact through the CSD with the IR *β*-subunit in rat adipose tissue [39]. *In vitro* studies showed that insulin stimulation of adipocytes promotes CAV1 Y14 phosphorylation and activation by IR kinase activity. Zimnicka et al. used advanced quantitative live-cell imaging to show that endocytosis and trafficking of caveolae are associated with conformational changes triggered by CAV1 Y14 phosphorylation. The latter spatially broadens CAV1 molecules within the oligomeric caveolar coat due to repulsion of adjacent negatively charged N-terminal phosphotyrosine residues. This spreading promotes caveolar inward bulging and endocytosis [40]. After CAV1 activation by IR, the cascade of signal transduction leads to the translocation of glucose transporter GLUT4 within caveolae [38, 41]. CAV1 is also implicated in the endocytosis of GLUT4 in insulin-stimulated adipocytes [42]. Equally, CAV1 silencing showed a reduction in insulin-induced GLUT4 recruitment to the cellular membrane and IR activation in adipocytes [43].

Insulin binding to IR causes a conformational change leading to activation of the intrinsic receptor tyrosine kinase that can phosphorylate IR on 13 potential tyrosines [44]. Signaling events downstream of IR autophosphorylation can be divided into two major pathways; the PI3K/AKT pathway, which controls the majority of insulin's metabolic actions, such as glucose uptake and glycogen storage, and the mitogenic or MAPK (mitogen-activated protein kinase) pathway, which governs cell growth and differentiation along with PI3K.

Insulin signaling follows different pathways and outcomes depending on the cell type [45]. Whether it is inducing gluconeogenesis in skeletal muscle cells or inhibiting lipolysis in adipocytes, the activation of IR starts in the caveolae and ends with it. The series of signaling events triggered by IR autophosphorylation is mediated by SHC and GRB2 (mitogenic pathway) or insulin receptor substrates (IRS) and SH2B2/APS (metabolic pathway). SHC and IRS interact with IR via their SH2 domain (Src homology 2) and are themselves tyrosine phosphorylated by IR [46, 47]. This IRS phosphorylation appears to be malfunctioning in human adipocytes and skeletal muscle obtained from patients with type 2 diabetes mellitus (T2DM) [48].

Autophosphorylation of IR also causes its internalization into an endosome mediated by an activated CAV1-P-Y14, which downregulates IR signaling activity [49]. Activated receptors get concentrated within the trafficked caveolar endosomes, and IR's tyrosine kinases can now phosphorylate substrates deemed inaccessible when caveolae were still attached to the cell surface. Endosomal proton pumps acidify the lumen, which leads to the insulin-IR uncoupling, and IR will either be degraded or dephosphorylated and recycled to the plasma membrane [50].

As aforementioned, CAV1—as an integral part of caveolae—is responsible for concentrating insulin receptors and some of the downstream effector proteins in caveolae and mediating their signaling effect, internalizing IR and then recycling it back to the cellular surface.

### 3.2. Role of Caveolin-1 in Insulin Resistance

*In vivo* disruption of caveolae results in insulin resistance. In humans, homozygous mutation in the *CAV1* gene causes CGL3, where affected patients have postprandial hyperinsulinemia, severe insulin resistance, hypertriglyceridemia, and lipodystrophy [51].

It was suggested earlier that adipose tissues show hypoxic areas causing the observed inflammatory response in the tissue [52], which may induce the development of T2DM as previously described. Recently, Varela-Guruceaga et al. investigated the role of CAV1 in hypoxia-induced insulin resistance in adipocytes. They observed a disruption in CAV1 localization in the plasma membrane of adipocytes, complete abolishment of the insulin-stimulated P-Y14 residues of CAV1, and a decrease in GLUT4 translocation to the plasma membrane suggesting CAV1 relevance in hypoxia-induced insulin resistance [53].

**Figure 2:**
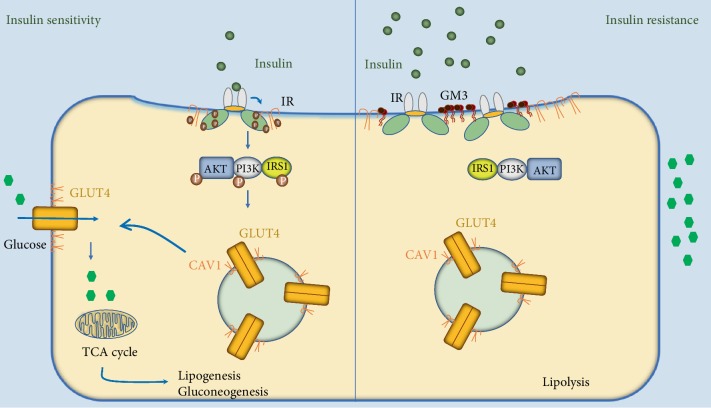
*During normal insulin sensitivity*, IR binds and activates CAV1 and then undergoes a series of autophosphorylations and activates the IRS/PI3K/AKT signaling pathway, which eventually leads to GLUT4 translocation in CAV1-mediated vesicles to the surface. *During insulin resistance*, while CAV1 and IR expression is increased, IR dissociates from CAV1 and moves into GM3-rich lipid rafts leading to impaired insulin signaling and decreased GLUT4 translocation to the membrane and causing a reduction in glucose uptake by the cell. Abbreviations: IR—insulin receptor; AKT—RAC-alpha serine threonine protein kinase; PI3K—phosphatidylinositol-3-kinase; IRS1—insulin receptor substrate 1; GLUT4—glucose transporter 4.

In fact, long-term exposure of adipocytes in culture to high glucose concentration increased CAV1 and IR expression and IR/IRS1/PI3K dephosphorylations and thus impairment of insulin signaling and insulin insensitivity [54]. In a different study, where adipocytes were induced by TNF-*α* to mimic a state of insulin resistance, live-cell experiments showed that IR was found to dynamically segregate from CAV1 in caveolae into glycosphingolipid GM3- (monosialodihexosylganglioside- or NANA-Gal-Glc-ceramide-) enriched microdomains compared to untreated adipocytes [55]. These results, taken together, suggest that during insulin resistance, the dissociation of IR from CAV1 leads to impaired insulin signaling and thus decreased GLUT4 translocation to the membrane, which reduces glucose uptake by the cell (see Figure 2). Blockade of the caveolae-mediated endocytosis of IR increased insulin signaling significantly in human adipocytes *in vitro* [56]. Mimicking this blockade can be a novel target for the treatment of T2DM insulin resistance.

## 4. Role of Caveolin-1 in Inflammation and Obesity

CAV1 expression in the adipose tissue is augmented in obese patients with or without T2DM. It was suggested in earlier reports that this could be due to the increased transport of fatty acids to the plasma membrane [57]. A recent study by Ghorpade et al. demonstrated that silencing of DPP-4 (dipeptidyl peptidase-4) in hepatocytes *in vitro* decreased insulin resistance and inflammation hallmarks in visceral adipose tissue. CAV1 siRNA had the same effect in adipose tissue macrophages (ATMs). CAV1 is, in fact, a mediator of the inflammatory effects of DPP-4 and plasma factor Xa (FXa) on lymphocytes [58]. Ghorpade et al. proposed a mechanism where obesity triggers a Ca^2+^-CaMKII-ATF4 (*calcium-*calmodulin-dependent protein kinase II-activating transcription factor 4) pathway in the liver, leading to DDP-4 secretion, which then stimulates a CAV1-IRAK1-TAK1 (interleukin-1 receptor-associated kinase1-TGF*β* activated kinase 1) pathway in ATMs, promoting ERK1/2-NF*κ*B-mediated inflammation. Visceral adipose tissue inflammation aggravates glucose intolerance, weakened insulin signaling, and hyperinsulinemia. The authors concluded that targeting this pathway might be more beneficial than using oral hypoglycemics [59].

Multiple lines of evidence have linked caveolae to the regulation of cholesterol homeostasis. Initially, caveolins are cholesterol-binding proteins and are involved in cellular sterol transport [60]. Furthermore, cellular cholesterol concentration is central for caveolae formation [61]. Depleting the cell membrane from its cholesterol has shown disappearance of surface caveolae structures, marked changes in CAV1 distribution, and impairment in cellular signaling [62]. CAV1-null mice had lower fat mass at older age, were resistant to HFD-induced obesity, had lower hepatic lipid accumulation, and had decreased metabolic flexibility compared to wild-type mice [63, 64]. These mice exhibit increased levels of serum triglycerides and free fatty acids and reduced levels of the cell surface fatty-acid binding protein (CD36) [65, 66]. Mechanistically, it was suggested that adipocyte lipid droplet size is diminished due to a defective fatty acid uptake through CD36 in adipocytes and fibroblasts [67]. More recent work showed that in human subjects enrolled in a trial of 8 weeks of diet-induced obesity, adipocyte expansion response correlated with initial CAV1 expression in the collected subcutaneous adipose tissue. Combining this result with other *in vivo* and *in vitro* work, the authors showed that CAV1 expression is crucial to increase caveolar density, improve adipocyte ability in accommodating larger lipid droplets, and promote cell expansion through ameliorated insulin response and improved glucose utilization [68].

Obesity causes adipocytes to become dysfunctional and secrete adipokines that recruit macrophages to the adipose tissue. These proinflammatory macrophages secrete cytokines such as TNF-*α* (tumor necrosis factor-alpha) and IL-6 (interleukin 6) [69] causing a chronic low-grade state of inflammation, which is linked to several diseases such as insulin resistance, diabetes, hyperinsulinemia, dyslipidemia, and vascular abnormalities [70]. Mechanistically, TNF-*α* signaling pathway and circulating free fatty acids cause ER stress responses that inhibit many of the insulin signaling downplayers such as IRS1/2, AKT, glycogen synthase, and glycogen phosphorylase, thus blocking glucose uptake and glycogen storage. Insulin sensitivity also correlated with P-Y14-CAV1 levels, which suggests a role for CAV1 in maintaining active and insulin-sensitive glucose uptake [71]. An *in vitro* study showed that overexpressing CAV1 in murine macrophages dramatically inhibited TNF-*α* and IL-6 production and increased the anti-inflammatory interleukin 10 secretion [72]. *In vivo*, CAV1 expression in monocytes from diabetic patients decreased, while TLR4 and TNF-*α* secretion increased and even more significantly in diabetic patients with neuropathy, suggesting, a role of CAV1 in regulating TLR4-mediated inflammatory cascade in T2DM [73].

The *CAV1* gene encompasses 36 kb and contains 3 exons producing 8 transcripts. The default transcript, which is representative of the biology and highly conserved, encodes an active 178 aa protein. Multiple long noncoding RNAs (*lncRNAs*) are transcribed upstream or downstream of the *CAV1* gene region with no known effect yet on *CAV1* gene regulation or expression. One particular lncRNA (AC006159.1) could be of interest for future investigation as it is transcribed from the reverse strand of the CAV1 intronic region.

*CAV1* can be regulated by different microRNAs. It has been demonstrated that miR-124a activates *CAV1* in podocytes, miR-375 downregulates *CAV1* gene expression [74], and miR-204 can target *CAV1* mRNA and decreases its expression in endothelial cells [75]. Trajkovski et al. identified *CAV1* as a target gene for miR103 and miR107. Their inactivation upregulates *CAV1* in adipocytes, thus improving IR maintenance, enhancing insulin signaling, reducing adipocyte size, and improving glucose uptake. The authors concluded that miR103/107 is important for insulin sensitivity and suggested it as a target for the treatment of T2DM [76].

**Figure 3:**
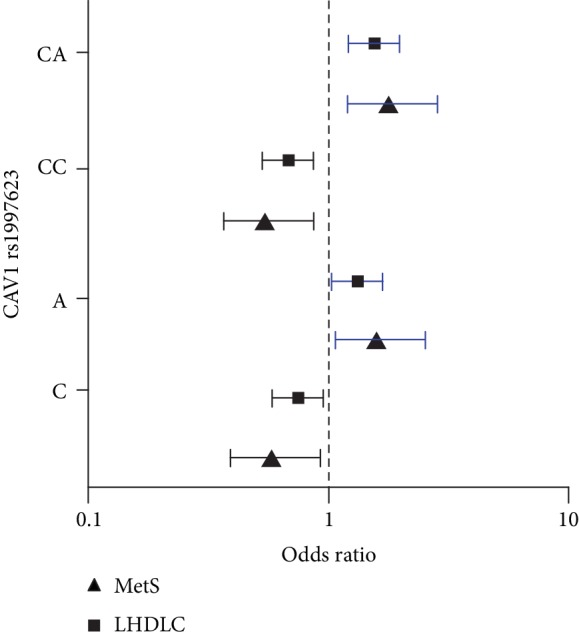
Forest plot representing relative odds ratio of MetS and LHDLC and their association with CAV1 rs1997623. CA—heterozygous; CC—wild type; A—A-allele; C—C-allele. The width of the horizontal lines represents the 95% confidence intervals for each measurement. Abbreviations: MetS—metabolic syndrome; LHDLC—low HDL cholesterol.

*CAV1* SNP variants have been suggested to be linked to metabolic syndrome (MetS), a major risk factor for diabetes and coronary artery disease. Several *CAV1* single-nucleotide polymorphisms (SNP) were found to associate with MetS: rs926198 in Caucasians and Hispanics [77], rs3807989 in the Chinese Han population [78], and rs1997623 in Kuwaiti children as shown by our previous study [79]. The forest plot in Figure 3 shows an association between LHDLC and MetS with the heterozygous genotype CA and the A-allele, meaning that a carrier of the A genotype has a higher chance of developing MetS in the studied population.

**Figure 4:**
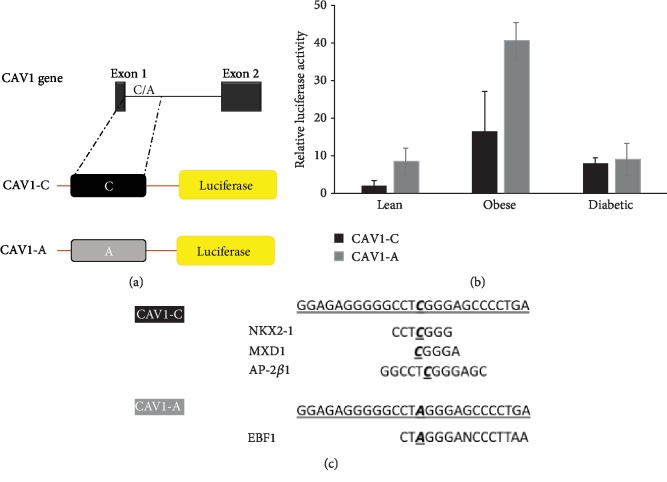
(a) Variants CAV1-C and CAV1-A cloning into the pRMT-Luc reporter vector. (b) Relative luciferase activity for pRMT-CAV1-C-Luc and pRMT-CAV1-A-Luc in lean, obese, and diabetic adipocytes. (c) Predicted binding sites for transcription factors depending on the CAV1-C and the sequence presenting rs1997623 SNP (CAV1-A). Abbreviations: NKX2-1—NK2 Homeobox 1; MXD1—MAX Dimerization Protein 1; AP-2*β*1—Adaptor-Related Protein Complex 2 Subunit Beta 1; EBF1—EBF Transcription Factor 1.

In order to study the role of the *CAV1* gene in MetS, we used the pRMT reporter vector (origene) to test the effect of rs1997623 polymorphism on promoter activity using luciferase as a reporter gene. We cloned 650 bp of the intronic region downstream of exon 1 (Figure 4(a)) containing the most common variant base C (CAV1-C) or the less common variant base A (CAV1-A) upstream of the luciferase gene. The plasmids were transfected into human primary adipocytes from lean, obese, and diabetic subjects. Predicted binding sites for transcription factors depending on sequence using the online tool Promo [80] are shown in Figure 4(c). Here, we show our primary results where luciferase activity is increased using the CAV1-A plasmid compared to CAV1-C in the obese adipocytes and not in the lean or diabetic adipocytes (Figure 4(c)). This result could be due to the possible loss of NKX2-1 (NK2 Homeobox 1), MXD1 (MAX Dimerization Protein 1), and AP-2*β*1 (Adaptor-Related Protein Complex 2 Subunit Beta 1) binding or the newly gained EBF1 (EBF Transcription Factor 1) binding that is affecting reporter gene transcription. It is noteworthy to mention that EBF1 is implicated in adipogenesis and the regulation of lipid metabolism by PPAR*α* (peroxisome proliferator-activated receptor alpha), while MXD1 is implicated in the MAPK-ERK pathway, apoptosis, and autophagy. CAV1 is known to play a role in the aforementioned metabolic pathways; further investigation is needed to possibly correlate MetS, *CAV1*, and these transcription factors. Another possibility could be that the rs1997623 variant affects an intronic regulator element lying downstream of exon 1 causing the increase in the luciferase activity in obese adipocytes and possibly be the reason behind the increased MetS and lower salivary HDL in Kuwaiti children harboring this variant.

## 5. Role of Caveolin-1 in Oxidative Stress

The expression of CAV1 is involved in cellular proliferation, senescence, differentiation, adhesion, and migration; nevertheless, the specific roles of CAV1 through these pathways remain unclear and they might change depending on the cell type [81–84]. In human breast cancer cells [85], CAV1 overexpression has antiproliferative activity and CAV1-null mutation in mice displayed epithelial and vascular hyperplasia [86]. CAV1 negatively regulates the ERK1/2 and PI3K/AKT signaling pathway [87], which leads to the upregulation of the cell cycle inhibitors p53 and p21 and the downregulation of the cell cycle promoter cyclin D1 causing a G0/G1 cell cycle arrest [88]. On the other hand, Fernandez et al. noticed that CAV1-null mice displayed weakened hepatic regeneration and impaired survival after partial hepatectomy. Hepatocytes were senescent and had drastically reduced lipid storage. Remarkably, glucose treatment of CAV1 KO mice increased survival and restored cell cycle progression. Therefore, the authors conclude that CAV1 is essential for liver regeneration and regulation of lipid metabolism [89].

**Figure 5:**
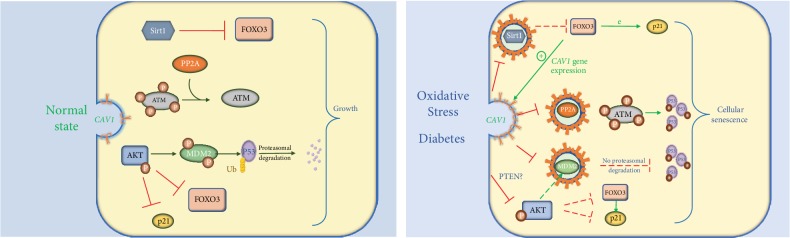
Simplified presentation of the role of CAV1 under oxidative stress and diabetes. During normal CAV1 expression, Sirt1 and p-AKT block p21 activator FOXO3. PP2A dephosphorylates ATM, thus attenuating p53 activation. Active AKT induces MDM2 activation, thus leading to the induction of p53 proteasomal degradation and ultimately normal cellular growth. During diabetes, CAV1 expression increases and caveolae sequester Sirt1, PP2A, and MDM2 preventing their actions, hence leading to the release of FOXO3, phosphorylation of p53 by ATM, and cell cycle arrest. CAV1 decreases p-AKT possibly by interacting with PTEN. The decrease in p-AKT reduces MDM2 activation and attenuates the deactivation of FOXO3 and p21, which eventually leads to cellular senescence. Abbreviations: Sirt1—Sirtuin 1; FOXO3—forkhead box protein O3; ATM—ataxia telangiectasia-mutated; PP2A—protein phosphatase 2A; AKT—RAC-alpha serine threonine protein kinase; MDM2—E3 ubiquitin ligase mouse double minute 2 homolog; p53—tumor protein p53.

Our previous results and others showed that CAV1 expression level increases in diabetes, oxidative stress, and senescent cells. We have found that CAV1 sequesters MDM2 (E3 ubiquitin ligase mouse double minute 2 homolog), which ubiquitinates p53. The latter would be targeted for proteasomal degradation resulting in cellular proliferation [90]. This sequestration causes p53 and p21 accumulation and the cells become senescent [84]. Furthermore, CAV1 inhibits Sirtuin 1 (Sirt1), which is a histone deacetylase that controls a plethora of physiological processes, including senescence. Oxidative stress induces seizing of Sirt1 into caveolae and the interaction of Sirt1 with CAV1 through its CSD, leading to inhibition of Sirt1 activity and activation of p53/senescence signaling [91]. Under physiological conditions, Sirt1 deacetylates forkhead box protein O3 (FOXO3), attenuates FOXO-induced apoptosis, and stops FOXO-p21-dependent cell cycle arrest [92]. Interestingly, transcription of the *CAV1* gene is directly stimulated by FOXO3 in a cell-cycle-independent manner; thus, the three factors CAV1, SIRT1, and FOXO3 are functioning in a feedback loop regulating cell cycle arrest [93–95]. CAV1 also activates ATM (ataxia telangiectasia-mutated) likely by sequestering the catalytic subunit of PP2A (protein phosphatase 2A) into caveolar structures, thereby stimulating engagement of the ATM-p53-p21 pathway [96] (Figure 5).

On the other hand, during normal or tumorigenic states, activated AKT phosphorylates MDM2 on two serine residues [97, 98], which promotes MDM2 nuclear localization and the subsequent p53 ubiquitination [99]. Post injury and in physiological conditions, high PTEN (phosphatase and tensin homolog) inhibits the PI3K/AKT signaling pathway and limits the proliferation of fibrotic tissue. Protein expression of both CAV1 and PTEN was diminished in lung fibroblasts in patients with idiopathic pulmonary fibrosis and in cardiac fibroblasts relative to control patients [100, 101]. Moreover, CAV1-null mice demonstrate low expression of PTEN compared to wild-type mice. CAV1 reconstitution in the knockout fibroblasts exhibited an elevated association of PTEN with the lipid bilayer and decreased PI3K/AKT signaling. Likewise, phosphorylated AKT is increased in liver and adipose tissue treated with CAV1 siRNA [59]. Altogether, CAV1 negatively modulates AKT signaling leading to decreased proliferation most likely by affecting PTEN expression levels. How CAV1 might be regulating PTEN is yet to be discovered.

## 6. Caveolin-1 in Chronic Complications of Diabetes

### 6.1. Caveolin-1 and Cardiovascular Disease in Diabetes

#### 6.1.1. Vascular Complications

Decreased insulin sensitivity strongly disturbs endothelial function and is the major contributor to the progression of diabetic macrovascular complications [102]. Endothelial dysfunction is categorized by impaired vasodilation in conduit and resistance arteries, which contributes to the genesis of hypertension, atherosclerosis, and coronary artery disease [13, 40, 103]. Studies in CAV1-deficient mice revealed that CAV1 can downregulate eNOS (endothelial nitric oxide synthase), which is primarily responsible for the generation of nitric oxide (NO) in the vascular endothelium, thus increasing paracellular permeability [104, 105]. Insulin exerts its vascular effects by activating eNOS and the release of NO [106].

The most important mechanisms controlling vascular function are gap junction communication between vascular cells and endothelial production of vascular modulators (NO), vasodilator prostaglandins such as prostacyclin (PGI2), and endothelium-dependent hyperpolarization (EDH) factors [107]. These mechanisms range from diameter and structure regulation to the protection against atherosclerosis. PGI2 is a vasodilator and a platelet activation inhibitor, NO and EDH factors regulate vascular tone of large conduit vessels and small resistance vessels, respectively, and their imbalance is at the basis of cardiovascular diseases [108].

Endothelial cells lacking CAV1 exhibit a rise in PGI2 expression indicating that CAV1 might be implicated in PGI2 expression regulation [109]. The key constituents of NO production and gap junctions, eNOS and connexins (Cx), respectively, are contained in caveolae and physically associate with CAV1 in human endothelial cells [110, 111]. *In vivo* and *in vitro* evidence show that NO increase weakens gap junction communication through channels with Cx37 [112, 113], and vice-versa, loss of connexins affects eNOS expression and function and disruption of caveolae affects both NO signaling and gap junction function [114]. In fact, CAV1 is essential for proper vascular connexins' localization (Cx37, Cx40, and Cx43) to the membrane in the aorta and superior mesenteric artery, formation of functional myoendothelial junctions, and adequate vasodilatory responses [110].

*(1) CAV1 and Coronary Disease*. Ablation of the CAV1 gene aggravates cardiac dysfunction and decreases survival in mice exposed to myocardial ischemia. Mechanistically, CAV1-/- mice subjected to coronary artery ligation display abnormalities in *β*-adrenergic signaling [115]. Recently, the cardioprotective ischemic preconditioning (IPC) was found to involve increased NO production, Cx43 phosphorylation, and chemical gap junction uncoupling [116]. Diabetes attenuates IPC by inhibiting CAV1; Gupta et al. were able to restore the protective IPC and increase NO production in rat diabetic heart [117].

In unstimulated endothelial cells, CAV1 inhibits NO production by binding to eNOS and suppressing its activity in the caveolae [118]. After stimulation, Ca^2+^-CaM binds to eNOS and CAV1 detaches to permit full activation of eNOS [119]. If CAV1 is knocked out, there is a loss of proper regulation of eNOS function [86]. Hyperlipidemia enhances CAV1/eNOS interaction and reduces endothelial NO production, thus contributing to endothelial dysfunction and atherosclerotic plaque formation [120, 121].

CAV1 expression is higher in EDH-mediated coronary microvessels than in NO-mediated conduit arteries in the control group. On the other hand, CAV1 was unaltered in coronary arterioles after ischemia. These results indicate that there are significant compensatory interactions between eNOS and CAV1 in diabetes *in vivo* and that EDH is involved in coronary vasodilatation after ischemia [122]. Nevertheless, excessive NO production can induce superoxide release and nitrooxidative stress and consequently counter NO bioavailability [123]. Hence, CAV1 can possibly maintain the endothelial function through its ability to regulate NO production under normal physiological conditions. In normal conditions, insulin activates eNOS located in the caveolae through the AKT pathway causing its phosphorylation on ser1177. *In vitro* hyperglycemia [124] and T2DM in humans result in the O-linked N-acetylglycosylation of Ser1177 [125], thus interfering with the signaling mechanisms of eNOS and attenuating NO production.

Insulin resistance, obesity, and MetS increase oxidized LDL levels in plasma. The latter was found to cause caveolar cholesterol depletion leading to decreased eNOS inactivation, while HDL maintains the caveolar lipid structure and preserves the capacity for eNOS to be activated. HDL has another antiatherogenic property by binding to SR-BI (scavenger receptor BI) in caveolae and inducing eNOS phosphorylation at ser1177 [126]. Alternatively, CAV1-deficient mice have elevated plasma HDL levels [66]. In fact, CAV1 associates to and enhances the internalization and degradation of ABCA1 (ATP-binding membrane cassette transporter A1) [127], a key protein in HDL synthesis. This is one of the mechanisms by which decreasing CAV1 expression can produce antiatherogenic effects.

As aforementioned, CAV1-null mice have impaired cholesterol homeostasis, insulin resistance, elevated NO production, and defects in cardiopulmonary and vascular function [19]. CAV1 and ApoE double-knockout mice are strongly protected against atherosclerotic lesions compared to ApoE-/- mice, despite elevated hypercholesterolemia and hypertriglycemia. The reexpression of CAV1 in the endothelium fully recovers atherosclerosis in ApoE-/- CAV1-/- mice [66]. Increased NO production due to CAV1 removal has long been thought to be the reason behind the atheroprotection observed in CAV1-/- mice. Surprisingly, Ramirez et al. very recently showed that this protection is done independently of eNOS activation and NO production. They have crossed CAV1-/- *with eNOS*-/- *mice* into a proatherogenic background and found that CAV1 controls proatherogenic responses of endothelial cells to disturbed blood flow, including lipid trafficking, extracellular matrix (ECM) remodeling, and inflammatory signaling in early-stage atherosclerotic lesions. CAV1 also promotes proatherogenic matrix deposition leading to endothelial cell activation in atheroprone regions of the aorta [128]. Hence, these data indicate that CAV1-based therapeutics should be further studied to alleviate the burden of diabetic complications.

*(2) CAV1 and Cerebrovascular Disease*. Diabetes increases the risk of cerebral ischemia either by ischemic stroke or cardiovascular diseases [129]. CAV1 KO mice ischemic brains showed impaired angiogenesis and increased apoptotic cell death [130]. CAV1 was later found to be downregulated in focal cerebral ischemia and reperfusion injury. In fact, CAV1 regulates the blood-brain barrier (BBB) permeability through modifying matrix metalloproteinases' (MMP) activity responsible for ECM destructuring [131]. CAV1 decreased cerebral infarct volume, facilitated angiogenesis and neurogenesis, and promoted neurological recovery by upregulating the VEGF signaling pathway in models of middle cerebral artery occlusion [132]. CAV1 expression is regulated by cystatin C during the maintenance of BBB integrity after ischemic brain injury. Lentiviral overexpression of CAV1 inhibited tight junction degradation and attenuated cerebral edema in rats [133]. In humans, cerebral edema is a dreaded complication of diabetic ketoacidosis in children [134]. These studies identify new potential therapeutic strategies for stroke.

*(3) CAV1 and Peripheral Artery Disease*. Angiogenesis is impaired in diabetes; CAV1-null mice fail to recover functional vasculature in hindlimbs after induced ischemia [135]. Recently, endothelial CAV1 was found to be central for EDH-mediated vasodilatation and ischemic angiogenesis through mediating protein S-nitrosylation by reactive nitrogen species (RNS) in a mouse model of hindlimb ischemia [136].

Type 1 diabetes (T1DM) and T2DM induce oxidative stress and increase CAV1 expression, which inhibits NO production and thus vasodilation, while CAV1-null mice showed improved arterial relaxation [135]. The decrease in NO bioavailability causes impairment of endothelium-dependent relaxations. However, EDH appears to compensate at least in part for this dysfunction in human subcutaneous arterioles with T2DM [137].

#### 6.1.2. Myocardial Impairment

Diabetic patients are at high risk of developing ventricular hypertrophy, and heart failure is the leading cause of death in diabetes. For a long time, it was thought that only caveolin-3 is expressed in the heart; however, CAV1 was later found to exist and function in cardiac myocytes [138], which explains why systemic CAV1-null mice exhibit striking biventricular hypertrophy with severely reduced systolic and diastolic heart function [139]. Although these mice show improved vascular responses [86], disruption of CAV1 leads to enhanced nitrosative stress [139] and eventual development of cardiomyopathy and pulmonary hypertension due to persistent eNOS activation, NO release, and p42/44 MAP kinase activation [140, 141]. Reexpression of CAV1 in the endothelium rescues the vascular and cardiac defects in CAV1-null mice [142], and phosphorylation of CAV1 was found to contribute to cardiac protection in isoflurane-induced mice [139].

Meta-analysis of prospective cohort and case-control studies of diabetes and risk of atrial fibrillation (AF) showed that diabetic patients have an increased risk of 40% to develop this major cause of thromboembolic stroke [143]. CAV1 is downregulated in atrial specimens of 13 patients with AF. Human atrial fibroblasts treated with CAV1 peptides abolished the profibrotic cytokine transforming growth factor-beta 1- (TGF-*β*1-) induced collagen production and decreased MMP2 and 9 expressions. Thus, the authors concluded that CAV1 is an important anti-AF signaling mediator by conferring its antifibrotic effects in atrium [144].

### 6.2. Caveolin-1 and Diabetic Nephropathy

Diabetic nephropathy (DN) is characterized structurally by mesangial expansion of the glomeruli, basement membrane thickening, and nodular glomerulosclerosis. Subsequently, interstitial fibrosis with tubular atrophy develops, along with arteriolar wall thickening, macrophage and T-lymphocyte infiltration, podocyte damage, and decreased endothelial cell fenestrations. Functionally, DN is characterized by glomerular hyperfiltration and increased albumin excretion, and in later stages by increasing proteinuria and declining glomerular filtration rate [145]. Many factors trigger these pathological changes such as hyperglycemia, mechanical stretch, oxidative stress, angiotensin II, chemokines, and profibrotic cytokines such as CTGF (connective tissue growth factor) and TGF-*β*1.

Caveolins play an important role in the regulation of diverse signaling pathways implicating the aforementioned triggers. Moriyama et al. assessed CAV1 expression in multiple glomerular diseases such as DN in humans. They have found augmented CAV1 expression and caveolae number in glomerular endothelial cells, which correlated with the degree of albuminuria [146].

In primary renal cells, CAV1 was found to be important for the trafficking of SGLT1 and SGLT2 (sodium/glucose cotransporters) to the cellular membrane, thus increasing glucose uptake by renal cells, indicating the importance of CAV1 in the pathology of glucose uptake in the kidney [147]. Caveolae and CAV1 enhance the biosynthesis and build-up of ECM in mesangial cells in response to elevated glucose and mechanical strain by promoting many profibrotic pathways [148–150]. CAV1 KO provided substantial protection from ECM accumulation and albuminuria in T1DM mice. They were considerably guarded from the rise of glomerular collagen I, CTGF, fibronectin, and TGF-*β*1 [151]. CAV1 was found to be necessary for glucose-induced generation of reactive oxygen species (ROS). Furthermore, caveolae are required for the activation of PKC*β* (phosphor kinase C beta), upstream of ROS, and eventually upregulating DN-associated TGF-*β*1 [152].

In the kidney, CAV1 mediates Ang II (angiotensin II) uptake in proximal tubules. This was functionally associated with diminished sensitivity to Ang II infusion in CAV1-null mice [153]. CAV1 acts as a chaperone for AT1R (Ang II type 1 receptor), helping its shuttling to the plasma membrane [154]. Nephrin is an important protein that signals apoptosis and consequently decreases renal cellular death; its dephosphorylation was detected in DN and was found to associate with podocyte impairment and loss [155]. In rats injected with Ang II, nephrin dephosphorylation was linked to CAV1 P-Y14 activation [156].

As in the other endothelial tissues, NO and eNOS were found to play a central role in the pathogenesis of DN. Indeed, CAV1 plays a pivotal role in the eNOS-mediated decrease of renal NO levels, which is possibly responsible for the progression of DN in T1DM and T2DM rat models [157].

There is a causal association of elevated homocysteine levels with the development of T2DM [158]. Recently Pushpakumar et al. showed that homocysteinylation of eNOS and disruption of caveolin-mediated regulation leads to ECM remodeling and hypertension in mice. H_2_S treatment attenuated renovascular damage by modulating eNOS and CAV1 interaction and thus antagonizing ECM protein accumulation and smooth muscle cell proliferation [159].

Combined data show further that targeting CAV1 can be a very effective therapeutic agent against DN.

### 6.3. Caveolin-1 and Diabetic Retinopathy

Diabetes causes several eye disorders such as diabetic retinopathy (DR), diabetic macular edema (DME), glaucoma, and cataracts. Early DR is characterized by the breakdown of the blood-retinal barrier (BRB). Diffuse retinal vascular permeability causes BRB loss and denotes a significant mechanism quickening the diabetes-mediated retinal changes [160]. In later stages, DME is caused by abundant vascular leakage, which leads to blindness in patients with DR, principally in T2DM patients with nonproliferative DR [161].

CAV1 constitutes a part of the rod cell outer segment, hence the possible role of CAV1 in ocular diseases [162]. In the retina, caveolar transcytosis and size were upregulated in streptozotocin- (STZ-) induced diabetic rats [10]. CAV1 was also found to be upregulated in rat diabetic retina [163]. During inflammatory conditions such as DR, CAV1 expression is dramatically increased in retinal Müller glial cells. Likewise, caveolae are increased in number and show bipolar localization in the pericytes (smooth muscle cells) and endothelial cells of the retinal neurovascular unit possibly promoting transcellular permeability [164].

Intravenous administration of advanced glycation end product- (AGE-) modified proteins in normoglycemic rats caused pathophysiological characteristics of DR such as retinal basement membrane thickening and loss of pericytes [165]. AGE-modified proteins can be internalized by caveolae in cultured retinal endothelial cells [166]. When injected into nondiabetic rats, they induce BRB loss, which was found to correlate with increased caveolar formation in the endothelium [167]. These results suggest that the augmented caveolae-mediated transendothelial permeability is a significant BRB failure player. Gu et al. in 2017 suggested that the increased CAV1 expression can potentiate Toll-like receptor signaling and proinflammatory cytokine release, which contribute to the BRB breakdown [164]. Unexpectedly, increased endothelial permeability was seen in CAV1 knockout mice [168]. This paradox can be explained by the vasodilation induced by the release of eNOS due to CAV1 loss, thus causing an augmentation in hydraulic pressure [169].

Proliferative vitreoretinopathy (PVR) is the foremost complication of severe DR and other ocular diseases. Its pathophysiology maps a very complex pathway ensuing a proliferative response within the retina [164]. CAV1 was recently found highly expressed in the proliferative membranes of mice eyes with PVR, and CAV1 reduction increased *retinal pigment epithelium* cell migration abilities. This indicates that CAV1 might be a potential therapeutic target in preventing proliferative membrane development in PVR [162].

Collectively, CAV1 needs to be overexpressed in knockout cells and decreased during inflammation for the protection against neovascularization or vitreoretinopathy. Further investigation in diabetic models is needed to delineate the effect of altering expression levels of CAV1.

### 6.4. Caveolin-1 and Diabetic Neuropathy

CAV1 is expressed in the neurons and glial cells of the central nervous system [170]. Classical caveolar endocytosis does not happen in neurons; there is a limited number of flask-shaped caveolae in the neuronal endothelium [171]. Nevertheless, targeted overexpression of CAV1 in neurons improves signaling, branching, and hippocampus-dependent learning and memory [172, 173]. During myelination, CAV1 is upregulated [174] and regulates cell signaling pathways in glial cells [175]. Chronic hyperglycemia decreases CAV1 expression in Schwann cells (SC) [176], while antisense downregulation of CAV1 in SC increased neuregulin-induced demyelination in hyperglycemic conditions (HGC) [177]. Furthermore, heightened brain lesion volume, neuroinflammation, and accelerated neurodegeneration were observed in CAV1-null mice [178, 179]. A relationship between CAV1 and Huntington disease was reported [180], and CAV1 was found to be a risk gene for schizophrenia [181]. All this implies that CAV1 may have a role in neural development. Several epidemiological data found an association between diabetes and lower cognitive test performance [182]. Experimental findings have shown significant cognitive impairment in STZ-induced diabetic rats [183]. CAV1 expression is impaired during prolonged HGC in cell lines and brain neurons of diabetic rats [184–186]. However, a link between CAV1 and diabetic neuropathies is not well established.

Another debated topic is the implication of diabetes in the etiology of sporadic Alzheimer's disease. CAV1-null mice exhibited several motor and behavioral anomalies and fast tau-related neurodegeneration such as Alzheimer's disease [172, 187]. Tau are microtubules binding phosphoproteins abundant in neurons [188]. Hyperphosphorylated tau is found in T2DM rat brains [189]. Wu et al. have found that chronic HGC reduce CAV1 expression and that CAV1-small interfering RNA (siRNA) increase tau phosphorylation and activate mTOR/S6K (mammalian target of rapamycin/S6 kinase) signaling in diabetic rat brain neurons. Likewise, overexpression of CAV1 decreases HGC-induced tau hyperphosphorylation in the hippocampus primary neurons [184]. Recently, lower CAV1 expression and higher amyloid-*β* levels were found in frozen brain sections of T2DM patients versus healthy subjects. The authors replicated the work in mice and found that the depletion of CAV1 in diabetic mice brains promotes neuropathology and impairments in learning and memory. CAV1 overexpression in these mice restored the learning deficiency [190]. Therefore, CAV1 might be an effective therapeutic target for diabetes-related cognitive decline.

Painful diabetic peripheral neuropathy (DPN) is a common complication of T2DM affecting almost 50% of diabetic patients and is very challenging to treat [155]. CAV1 seems to have a regulatory role in DPN development as diabetic CAV1-null mice developed a higher deficit in motor nerve conduction velocity and thermal and mechanical sensitivity when compared with diabetic wild-type mice [191]. Yang et al. showed that the knockdown or blocking of CAV1 in the murine brain reversed behavioral and neuronal sensitization of induced pain, while the overexpression of CAV1 caused pain behavior in the unaffected mouse paw. This modulation of neuropathic pain is done via regulation of NR2B (NMDA receptor 2B subunit) and subsequent activation of ERK/CREB signaling (cAMP response-element binding), suggesting a potent CAV1-based therapy for chronic neuropathic pain [192]. It was further demonstrated that the CAV1-NR2B pathway is activated by microglial JAK2-STAT3 signaling (Janus kinase 2-signal transducer and activator of transcription 3), which contributes to diabetic neuropathic pain. Pain was also relieved by administering AG490 (JAK2 inhibitor) to the rats [193]. Collectively these data render promising DPN therapies based on the JAK2-STAT3-CAV1-NR2B signaling pathway.

## 7. Caveolin-1 and Antidiabetic Drugs

The interaction of CAV1 with antidiabetic drugs (summarized in Table 1) has been mainly investigated in the context of different types of cancers. Currently GLP-1R agonist, metformin, sulfonylureas, DPP-4, and SGPTL2 inhibitors are the major class of oral drugs used for glycemic control in T2DM. Glucagon-like receptor (GLP-1R) agonists function by stimulating the adenylyl cyclase pathway that leads to increased synthesis and release of insulin. Previous studies have shown that membrane localization and binding activity of GLP-1R require its interaction with CAV1, and this is in turn influenced by specific mutations within the putative region of CAV1 or GLP-1R [194]. The overexpression of CAV1 breast cancer-associated mutant, P132L, results in the attenuation of GLP-1 binding activity, regulating the downstream cellular trafficking and signaling activity of GLP-1R [194]. Metformin functions by diminishing the hepatic glucose production and intestinal glucose absorption. Metformin causes an increase in the AMP/ATP ratio and adenosine monophosphate-activated protein kinase (AMPK) activation. Knockdown of CAV1 in bovine aortic endothelial cells has been reported to diminish VEGF-dependent AMPK activation [195]. Accordingly, CAV1 was found to contribute to the inhibitory action of metformin on insulin growth factor 1 (IGF-1) activity in non-small-cell lung cancer cells where their sensitivity to metformin was dependent on CAV1 expression and CAV1 was required to induce AMPK phosphorylation and the increase in the AMP/ATP ratio [196]. Metformin induces CAV1 expression [197], which in return increases clinical efficacy of drug delivery (trastuzumab emtansine) in breast cancer cells [198]. Sulphonylureas on the other hand increase insulin release by blocking K_ATP_ channels [199]. Glimepiride, a second generation sulphonylurea, alters plasma membrane dynamics resulting in CAV1 tyrosine phosphorylation [200]. CAV1 coimmunoprecipitates with Kir6.1 (a subunit of the K_ATP_ channel) in rat aortic cells and with Kir6.2 in the pancreatic *β*TC-6 cell line. *β*TC-6-CAV1-depleted cells maintained a high rate of insulin secretion after KCl, but not after glucose and glimepiride stimulation. These results combined suggest that CAV1 might play a role in sulfonylurea-stimulated insulin secretion maybe through regulation of K_ATP_ channels [201, 202]. Dipeptidyl peptidase-4 (DPP-4) inhibitors are yet another class of antidiabetic drugs that act by increasing intestinal incretins GLP-1 (glucagon-like peptide-1) and GIP (glucose-dependent insulinotropic polypeptide), leading to reduced glucagon release and increased insulin secretion. DPP-4 interacts with CAV1 in antigen-presenting cells [203] resulting in CAV1 phosphorylation and subsequent activation of the NF*κ*B pathway [58]. CAV1 has been reported to be a drug target for linagliptin. This DPP-4 inhibitor increases eNOS availability by blocking its binding with CAV1 [204] leading to enhanced NO production in mesenteric arteries from type 1 diabetic rats [205]. The interaction of CAV1 with the sodium-glucose cotransporter 1 and 2 (SGLT1 and SGLT2) inhibitors is not well documented in the literature. SGLT inhibitors improve glycemic control by inhibiting the renal glucose reabsorption allowing excess sugar to be removed through urine. As aforementioned, cAMP-stimulated SGLT trafficking in renal proximal tubules depends on CAV1 expression and intact actin filaments. Coinhibition of both CAV1 and F-actin blocks SGLT-mediated glucose uptake [147]. Another study showed that CAV1 increases the expression, activity, and transport of SGLT1 to the cell membrane in *Xenopus* oocytes [206]. However, a new study showed that SGLT1 internalization is lipid raft-dependent but CAV1-independent in HEK and COS cells [207].

Though CAV1 tends to improve glycemic control and associated complications via its interaction with various drug targets and downstream signaling molecules, its potential use as an antidiabetic drug requires further in-depth studies.

## 8. Future Perspectives

By virtue of its ability to affect numerous cellular pathways, CAV1 represents a challenging but interesting therapeutic target for micro- and macrovascular complications of diabetes. Since CAV1 can regulate several signaling cascades, targeting CAV1 to reduce inflammation and oxidative stress can be more efficient than aiming at the different pathways separately. Adding to this, the fact that CAV1 deletion is not lethal in mice, CAV1-based therapies can be a very enticing intervention for treating diabetic complications.

In the context of atherosclerosis, Sharma et al. have previously investigated the therapeutic effect of a cell-permeable CAV1-derived peptide, CavNOxin, on the development of atherosclerosis in T1DM model (STZ, ApoE-/-) and western diet-induced T2DM model mice. CavNOxin controlled oxidative stress and lowered diabetes-induced atherosclerotic plaque up to 84% *in vivo*. Mechanistically, CavNOxin attenuated oxidative stress markers, blocked leukocyte-endothelial interactions, and inhibited the expression of proinflammatory mediators, while preserving caveolar structures. Hence, CavNOxin should be further studied as a pharmacological therapy to alleviate the burden of diabetic macrovascular complications in both T1DM and T2DM [208].

Apropos of the treatment of nephropathy, many groups treated diabetic mice with different drugs that affect CAV1 expression, CAV1 activation (P-Y14), or caveolae formation such as salidroside, daidzein, curcumin, cyclodextrin, CSD, and PP2; they all mitigated nephropathy by at least decreasing proteinuria and ECM accumulation. For further information about these CAV1-based therapies, please read the beautiful review written by Krieken and Krepinsky [209]. An additional biomolecule called taxifolin (or dihydroquercetin (DHQ)) might have a promising effect on DN. Recent work showed that DHQ has protective kidney properties similar to losartan (angiotensin II inhibitor) including attenuating albuminuria, hyperglycemia, and lipid metabolism disorders, and modifying renal lesions in DN possibly by suppressing ROS and NLRP3 (NOD-, LRR-, and pyrin domain-containing protein 3) inflammasome [210]. Another study found that DHQ attenuates DN in STZ diabetic rats; CAV1 and p-NF*κ*B expression was reduced upon DHQ administration, which suggests that DHQ might have its beneficial effects on DN by downregulating CAV1 [211].

With respect to ischemic stroke, several natural compounds, including calycosin-7-O-*β*-D-glucoside, baicalin, *Momordica charantia* polysaccharide, chlorogenic acid, lutein, and lycopene, have shown potential for targeting the RNS/CAV1/MMP signaling pathway (see the review in [212] and references therein).

In regard to ocular diseases, Jiang et al. in 2017 found that CAV1 deficiency exacerbates ocular neovascularization, which ultimately causes vision loss in several pathological visual diseases such as DR. The authors used cavtratin, a lipophilic peptide of CAV1 CSD, to inhibit neovascularization. They found that the combined administration of cavtratin and anti-VEGF-A (the usual treatment for DR) inhibited neovascularization more effectively than monotherapy. Cavtratin exerts its effect by downregulating eNOS expression and the platelet-derived growth factor-B (PDGF-*B) in vivothrough the JNK pathway* [213]. These two factors play important roles in pathological angiogenesis [214, 215]. This CAV1 peptide may thus have promising therapeutic applications for the treatment of DR by targeting angiogenic factors. Conversely, the *in vivo* silencing of CAV1 by siRNA in the retina can suppress neovascularization and pathological BRB breakdown in ischemia-induced retinal disease [216]. It is noteworthy to mention that thiazolidinediones or peroxisome proliferator-activated receptor-gamma (PPAR*γ*) agonists that are used to treat T2DM, can suppress retinal neovascularization, attenuate retinal vascular inflammation and BRB breakdown, and promote neuroprotection in the retina [217, 218]. PPAR*γ* activation induces CAV1 [219] and has all the hallmarks of CAV1 action such as the previously mentioned decrease of insulin resistance, adipocyte regulation, and antiproliferative action [220]. A very recent study found that rosiglatizone can protect BBB by attenuating inflammation through a CAV1-depedent pathway. It is yet to be studied in the state of diabetes [221].

Decreasing CAV1 expression in a targeted manner is an important issue to consider as different conditions need different CAV1 protein levels. For instance, CAV1 expression in the brain decreases with age and the effect of silencing CAV1 might be detrimental to cognitive function [179]. Taking this into consideration, engineering a drug based on CAV1 should be formulated in a way to be impermeable through the blood-brain barrier if the desired outcome is downregulating the expression of CAV1 such as in the case of atherosclerosis and nephropathy.

## Figures and Tables

**Table 1:** Antidiabetic drugs and their relationship with CAV1.

Class	Effect on insulin	Drugs modulating CAV1	Relation with CAV1	Model/cell lines	References
Glucagon-like receptor (GLP-1R) agonists	Increase insulin synthesis and release	Not known	GLP-1R is internalized via CAV1-dependent mechanism	HEK and MIN6 cells	[194]
Metformin	Decreases hepatic glucose production and intestinal glucose absorption	Metformin	Increases CAV1 expression	Calu-1/6 and MCF-7	[196, 197]
Sulphonylureas	Stimulate insulin secretion	Glimepiride	Induce CAV1 tyrosine phosphorylation	Primary rat adipocytes	[200]
Dipeptidyl peptidase-4 (DPP-4) inhibitors	Slow the inactivation and degradation of GLP-1 and GIP	Linagliptin	Block CAV1 binding to eNOS	NOD mice aorta and HEK cells	[204, 205]
Sodium-glucose transport (SGLT) inhibitors	Inhibit renal glucose reabsorption	Not known	SGLT needs CAV1 for trafficking and expression?	Primary rabbit proximal tubule cells, *Xenopus* oocytes	[147, 206, 207]
